# Supplementary material for: Transport and inhibition mechanism for VMAT2-mediated synaptic vesicle loading of monoamines
Source: Cell Res. 2024 Jan 2;34(1):47–57. doi: 10.1038/s41422-023-00906-z (PMC10770148; doi:10.1038/s41422-023-00906-z)
Supplement: Supplementary file 1 — Supplementary information, Fig S1 [file 41422_2023_906_MOESM1_ESM.docx]

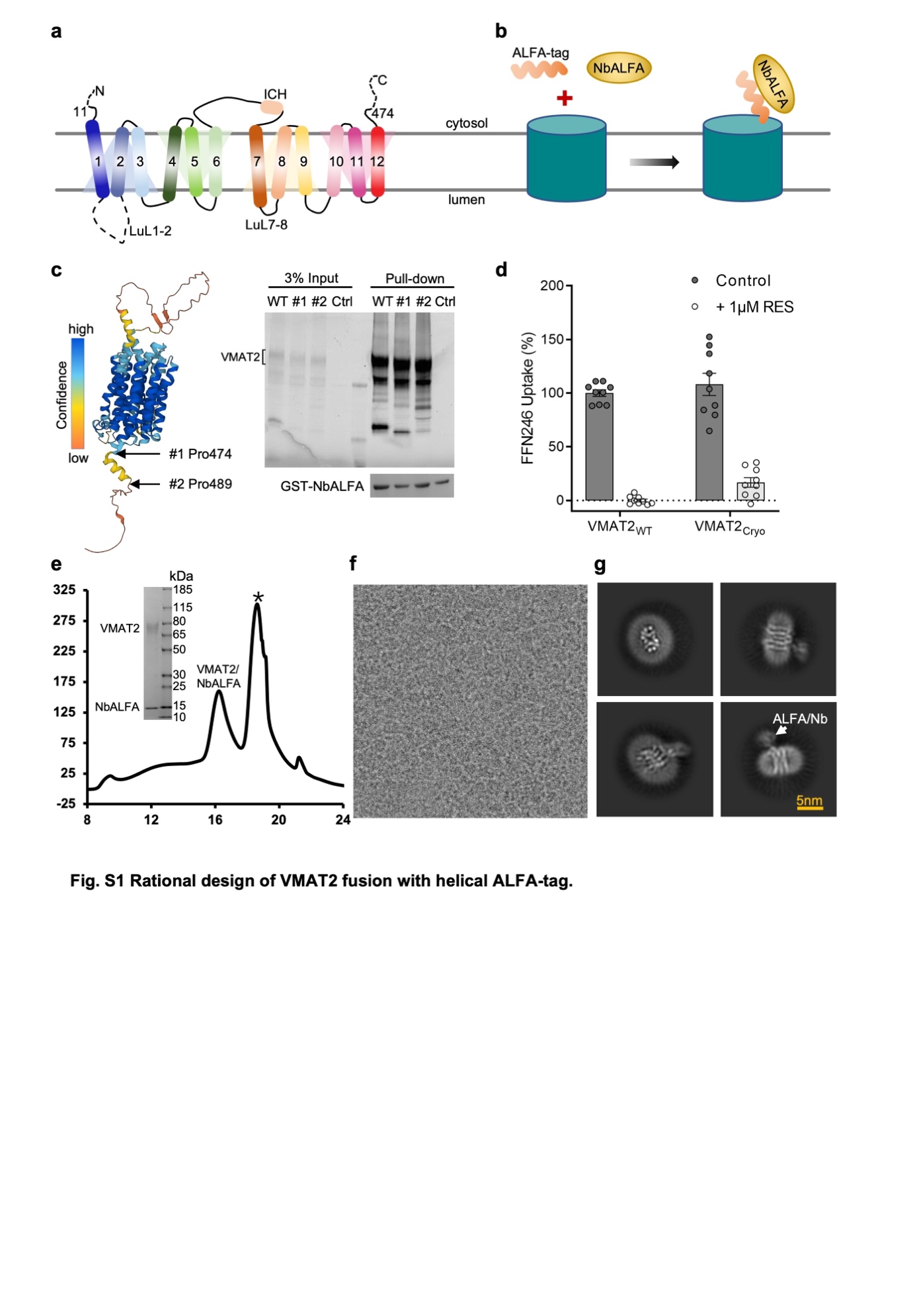


**Fig. S1 Rational design of VMAT2 fusion with helical ALFA-tag. a** Topology diagram of full-length human VMAT2 used in the study. Regions with ill-defined densities are depicted by dashed lines. **b** Schematic of ALFA-tag fusion and its high-affinity nanobody NbALFA as a fiducial marker for structual determination of small size membrane protein. **c** Rational engineering based on VMAT2 AlphaFold model, with binding confirmed by pull-down assay visualized via in-gel fluorescence. **d** Cellular uptake of FFN246 and inhibition for the wild-type VMAT2 (VMAT2_WT_) and the cryo-EM construct VMAT2_1-474_-ALFA co-expressed with NbALFA (VMAT2_Cryo_). For comparison, 1 mM reserpine (RES) was added to inhibit FFN246 uptake, and the 100% uptake activity was defined as the average uptake efficiency of VMAT2_WT_ without reserpine. Data are shown as the mean ± SEM; *n* = 3 biological repeats. **e** Size exclusion chromatography profile and SDS-PAGE result (inset) of VMAT2-ALFA/NbALFA sample. **f** Representative raw image of VMAT2 in thin vitrified ice. **g** Representative 2D class averages of VMAT2 particle alignment facilitated by ALFA-tag/NbALFA marker (indicated by white arrow).
