# Supplementary material for: Transport and inhibition mechanism for VMAT2-mediated synaptic vesicle loading of monoamines
Source: Cell Res. 2024 Jan 2;34(1):47–57. doi: 10.1038/s41422-023-00906-z (PMC10770148; doi:10.1038/s41422-023-00906-z)
Supplement: Supplementary file 2 — Supplementary information, Fig S2 [file 41422_2023_906_MOESM2_ESM.docx]

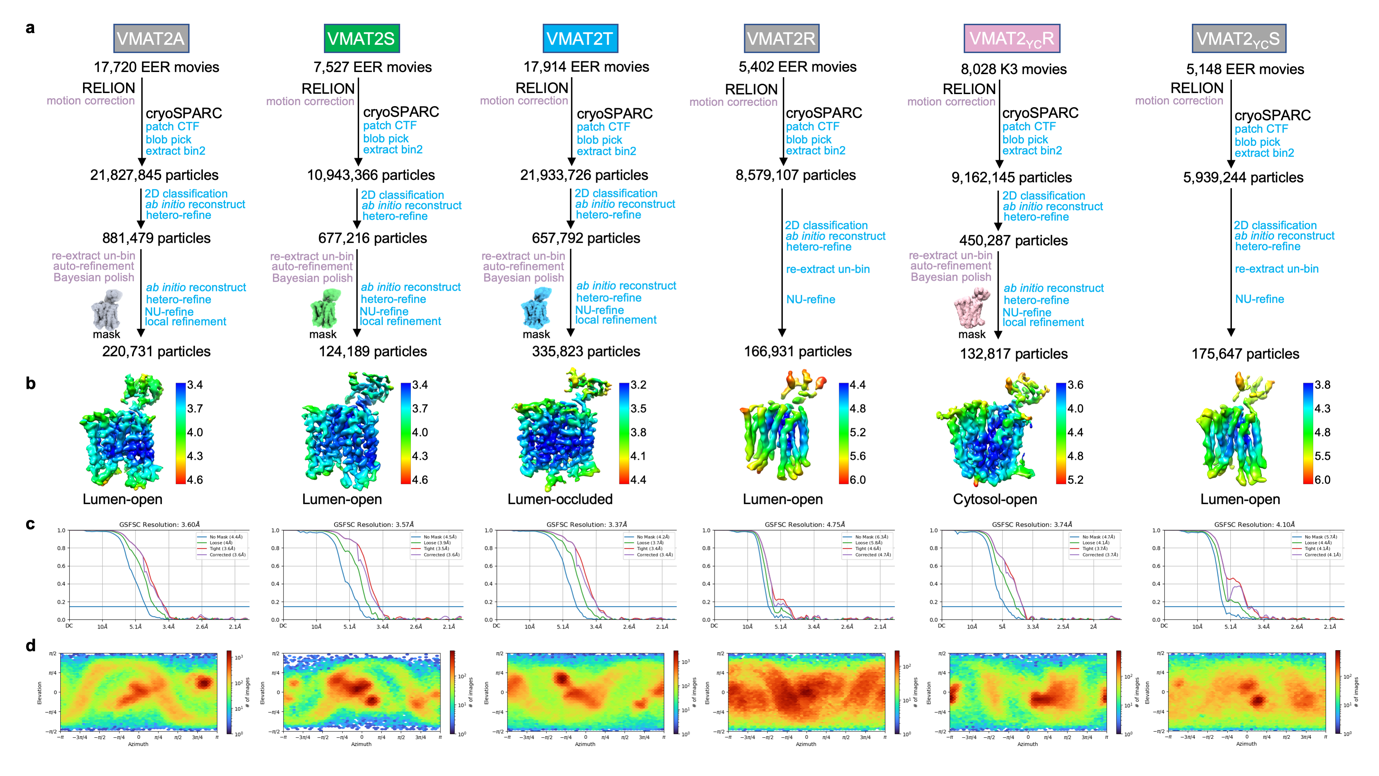
 **Fig. S2 Cryo-EM analysis of VMAT2 with different ligands. a** Processing workflow for VMAT2 wildtype protein in apo (VMAT2A), complex with substrate serotonin (VMAT2S), non-competitive inhibitor tetrabenazine (VMAT2T) and competitive inhibitor reserpine (VMAT2R), and VMAT2 Y422C mutant with reserpine (VMAT2_YC_R) and serotonin (VMAT2_YC_S). **b** Final reconstructed maps colored by local resolution, with conformational states indicated below. **c** Map resolution estimated by the gold-standard Fourier shell correlation (GSFSC) with cutoff at 0.143. **d** Angular distribution heatmap of the final particles used in the corresponding map reconstructions.
