# Supplementary material for: Transport and inhibition mechanism for VMAT2-mediated synaptic vesicle loading of monoamines
Source: Cell Res. 2024 Jan 2;34(1):47–57. doi: 10.1038/s41422-023-00906-z (PMC10770148; doi:10.1038/s41422-023-00906-z)
Supplement: Supplementary file 3 — Supplementary information, Fig S3 [file 41422_2023_906_MOESM3_ESM.docx]

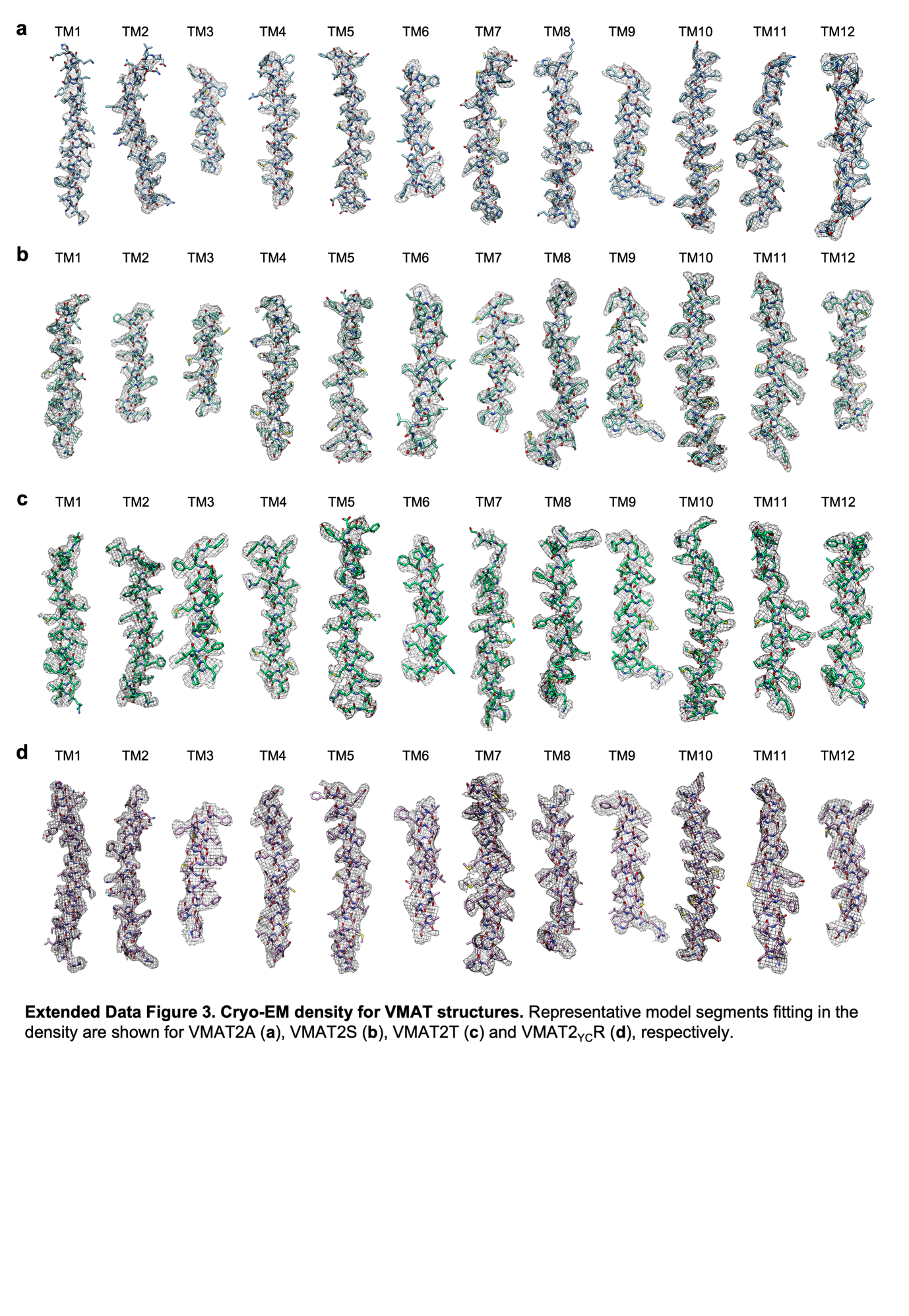


**Fig. S3 Cryo-EM density for VMAT structures.** Representative model segments fitting in the density are shown for VMAT2A (**a**), VMAT2S (**b**), VMAT2T (**c**) and VMAT2_YC_R (**d**), respectively.
