# Supplementary material for: Transport and inhibition mechanism for VMAT2-mediated synaptic vesicle loading of monoamines
Source: Cell Res. 2024 Jan 2;34(1):47–57. doi: 10.1038/s41422-023-00906-z (PMC10770148; doi:10.1038/s41422-023-00906-z)
Supplement: Supplementary file 4 — Supplementary information, Fig S4 [file 41422_2023_906_MOESM4_ESM.docx]

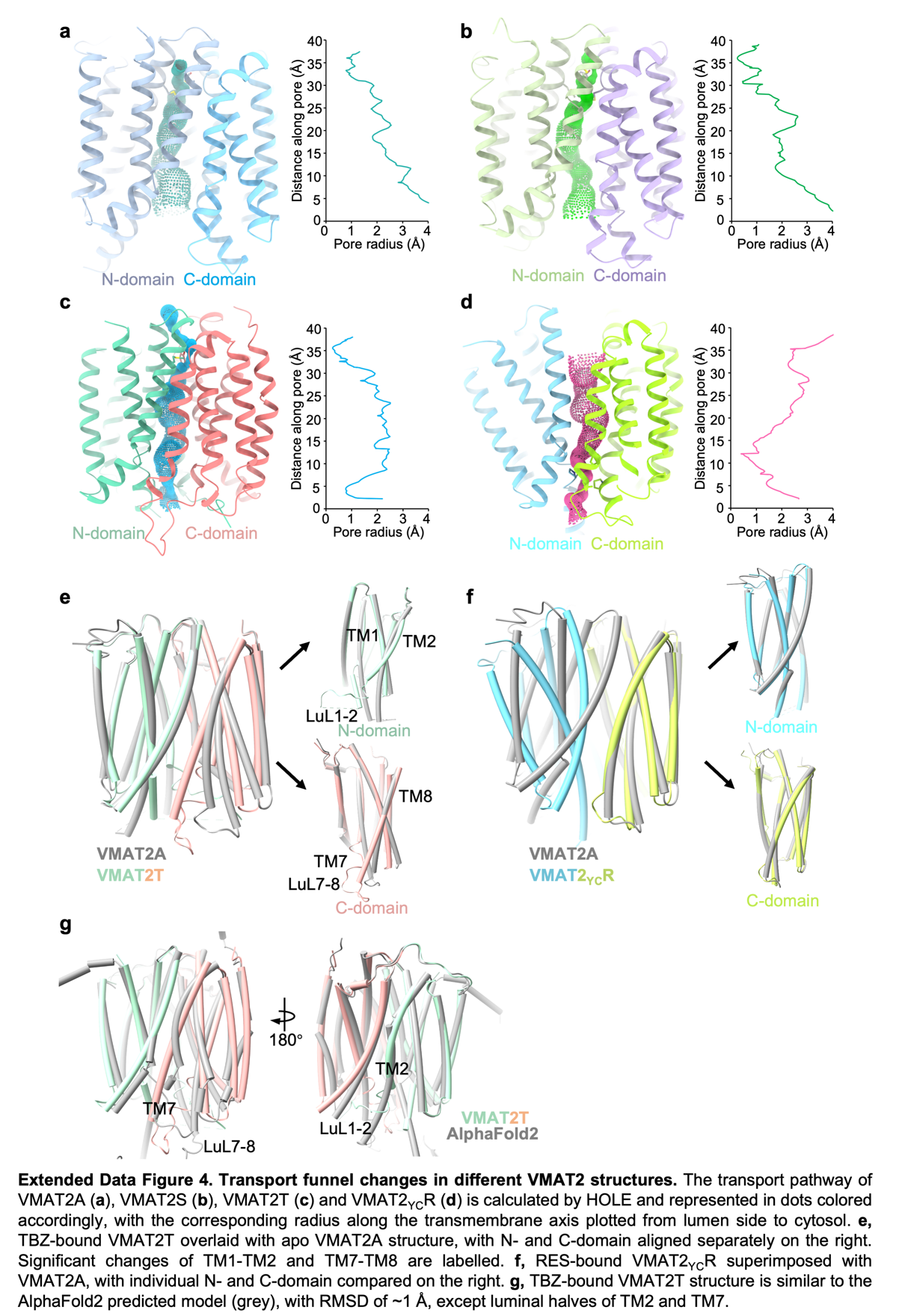


**Fig. S4 Transport funnel changes in different VMAT2 structures.** The transport pathway of VMAT2A (**a**), VMAT2S (**b**), VMAT2T (**c**) and VMAT2_YC_R (**d**) is calculated by HOLE and represented in dots colored accordingly, with the corresponding radius along the transmembrane axis plotted from lumen side to cytosol. **e,** TBZ-bound VMAT2T overlaid with apo VMAT2A structure, with N- and C-domain aligned separately on the right. Significant changes of TM1-TM2 and TM7-TM8 are labelled. **f,** RES-bound VMAT2_YC_R superimposed with VMAT2A, with individual N- and C-domain compared on the right. **g,** TBZ-bound VMAT2T structure is similar to the AlphaFold2 predicted model (grey), with RMSD of ~1 Å, except the unwound luminal halves of TM2 and TM7.
