# Supplementary material for: Transport and inhibition mechanism for VMAT2-mediated synaptic vesicle loading of monoamines
Source: Cell Res. 2024 Jan 2;34(1):47–57. doi: 10.1038/s41422-023-00906-z (PMC10770148; doi:10.1038/s41422-023-00906-z)
Supplement: Supplementary file 5 — Supplementary information, Fig S5 [file 41422_2023_906_MOESM5_ESM.docx]

**
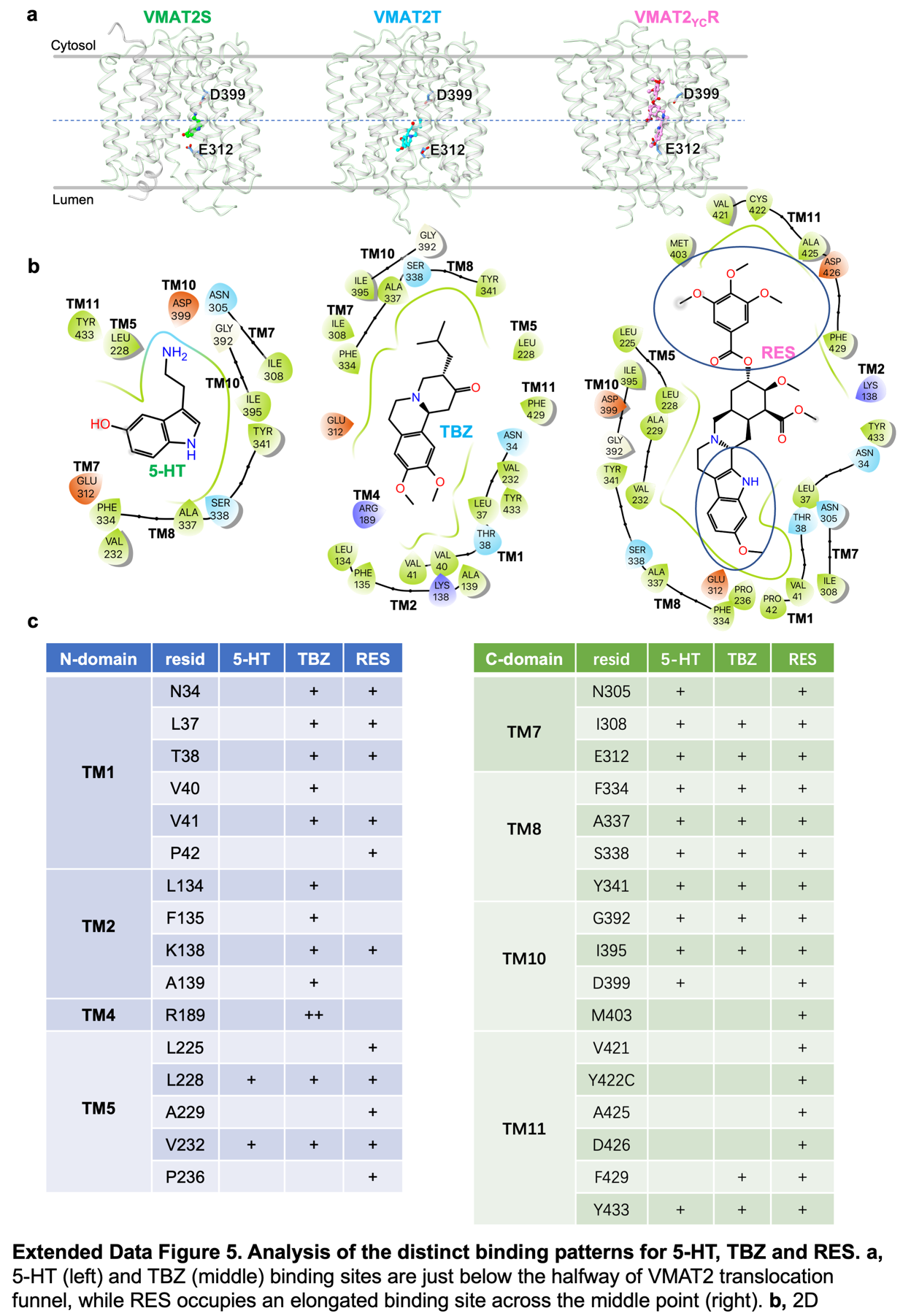
**

**Fig. S5 Analysis of the distinct binding patterns for 5-HT, TBZ and RES. a,** 5-HT (left) and TBZ (middle) binding sites are just below the halfway of VMAT2 translocation funnel, while RES occupies an elongated binding site across the middle point (right). **b,** 2D interaction diagram for residues interacting with 5-HT (left), TBZ (middle) and RES (right), analyzed in Maestro with a distance cutoff at 3.8 Å. Residues in green, red and blue correspond to hydrophobic, negative and positive charged features. **c**, Distribution of interaction residues in N- and C- domain of VMAT2 according to the analysis results in (**b**).
