# Supplementary material for: Transport and inhibition mechanism for VMAT2-mediated synaptic vesicle loading of monoamines
Source: Cell Res. 2024 Jan 2;34(1):47–57. doi: 10.1038/s41422-023-00906-z (PMC10770148; doi:10.1038/s41422-023-00906-z)
Supplement: Supplementary file 6 — Supplementary information, Fig S6 [file 41422_2023_906_MOESM6_ESM.docx]

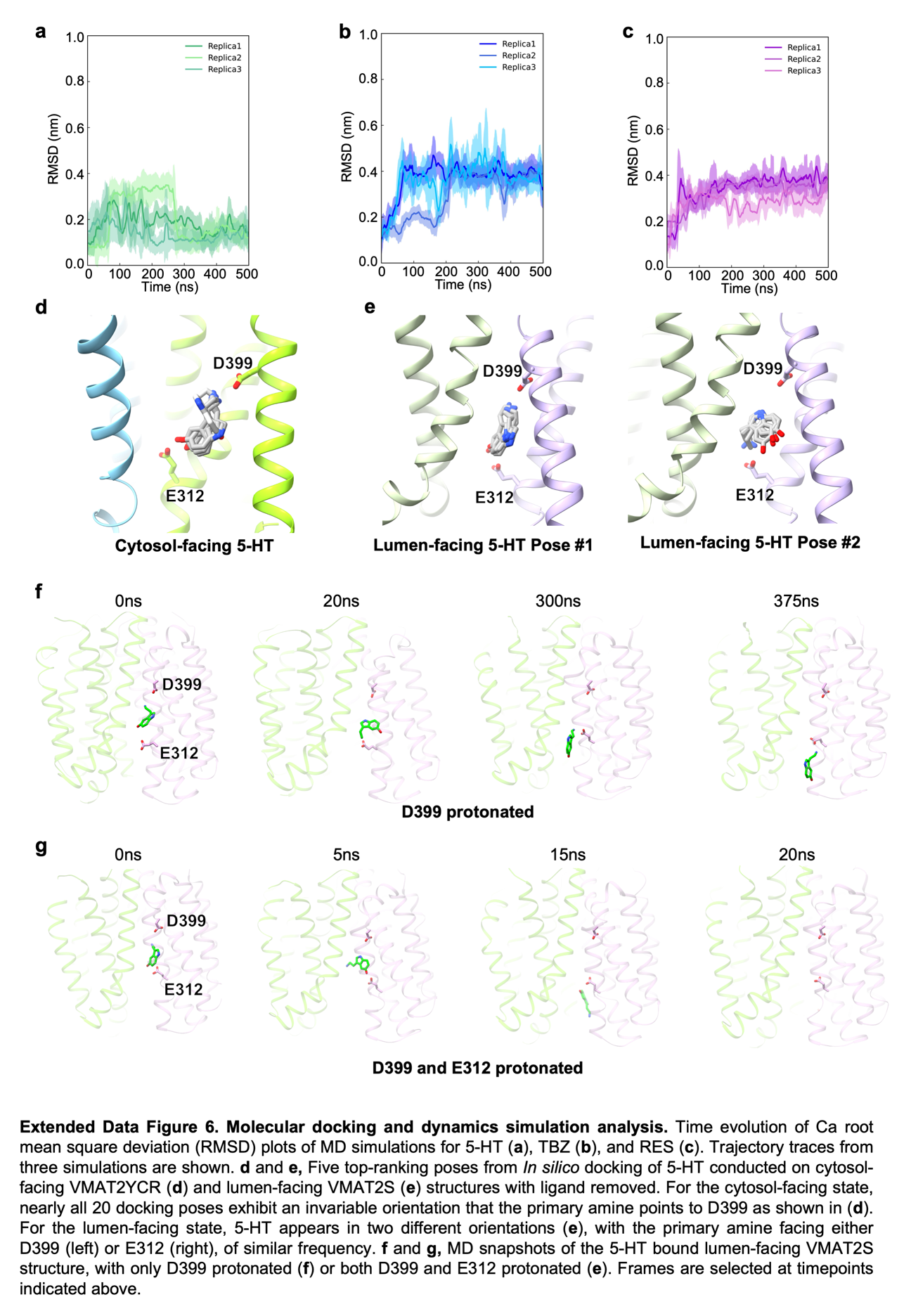


**Fig. S6 Molecular docking and dynamics simulation analysis.** Root mean square deviation (RMSD) plots of MD simulations for 5-HT (**a**), TBZ (**b**), and RES (**c**). Trajectory traces from three simulations are shown. **d** and **e,** Five top-ranking poses from *in silico* docking of 5-HT conducted on the cytosol-facing VMAT2_YC_R (**d**) and the lumen-facing VMAT2S (**e**) structures. For the cytosol-facing state, nearly all 20 docking poses exhibit an invariable orientation that the primary amine points to D399 as shown in (**d**). For the lumen-facing state, 5-HT appears in two different orientations (**e**), with the primary amine facing either D399 (left) or E312 (right), of similar frequency. **f** and **g,** MD snapshots of the 5-HT bound lumen-facing VMAT2S structure, with only D399 protonated (**f**) or both D399 and E312 protonated (**e**). Frames are selected at timepoints indicated above.
