# Supplementary material for: Transport and inhibition mechanism for VMAT2-mediated synaptic vesicle loading of monoamines
Source: Cell Res. 2024 Jan 2;34(1):47–57. doi: 10.1038/s41422-023-00906-z (PMC10770148; doi:10.1038/s41422-023-00906-z)
Supplement: Supplementary file 7 — Supplementary information, Fig S7 [file 41422_2023_906_MOESM7_ESM.docx]

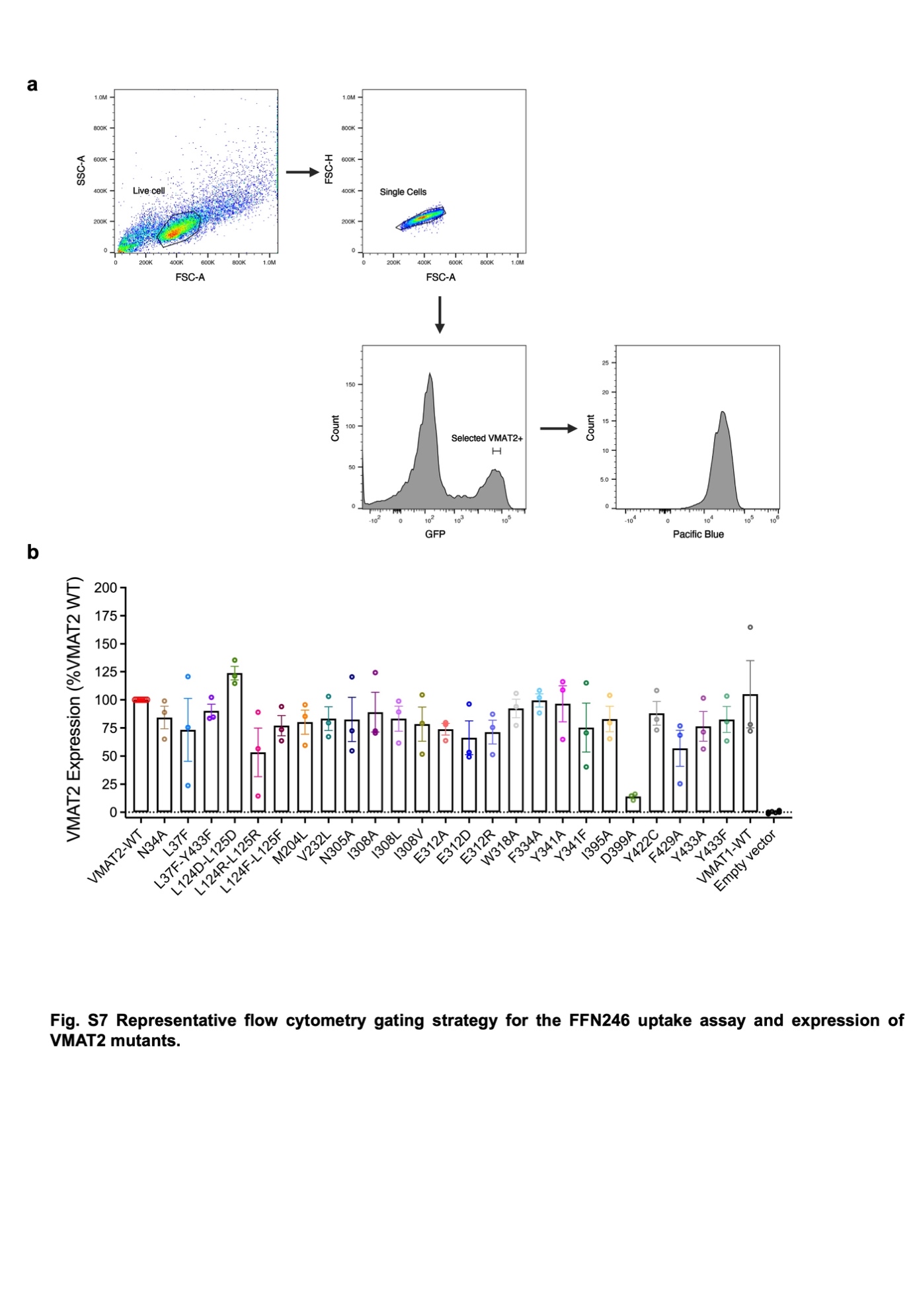


**Fig. S7 Representative flow cytometry gating strategy and protein expression levels for the FFN246 uptake assay. a** The cells were first gated by FSC and SSC to filter alive cells and single cells. The cells were then gated by GFP fluorescence to select the cells expressing VMAT2 or its mutants. Only events located in a small range of the GFP fluorescence signal were selected to make sure the protein expression level keeping approximately same in the different variants of VMAT2. The selected GFP^+^ subpopulation was then analyzed for FFN246 uptake level by violet laser channel 405/450. **b** Expression levels of VMAT2 mutants. The GFP fluorescence intensities of mutants were normalized to that of wildtype VMAT2. Except a reduced expression for D399A mutant, most variants exhibited comparable level as wildtype VMAT2 (mean ± SEM, *n*=3 biological replicates).
