# Supplementary material for: Transport and inhibition mechanism for VMAT2-mediated synaptic vesicle loading of monoamines
Source: Cell Res. 2024 Jan 2;34(1):47–57. doi: 10.1038/s41422-023-00906-z (PMC10770148; doi:10.1038/s41422-023-00906-z)
Supplement: Supplementary file 8 — Supplementary information, Fig S8 [file 41422_2023_906_MOESM8_ESM.docx]

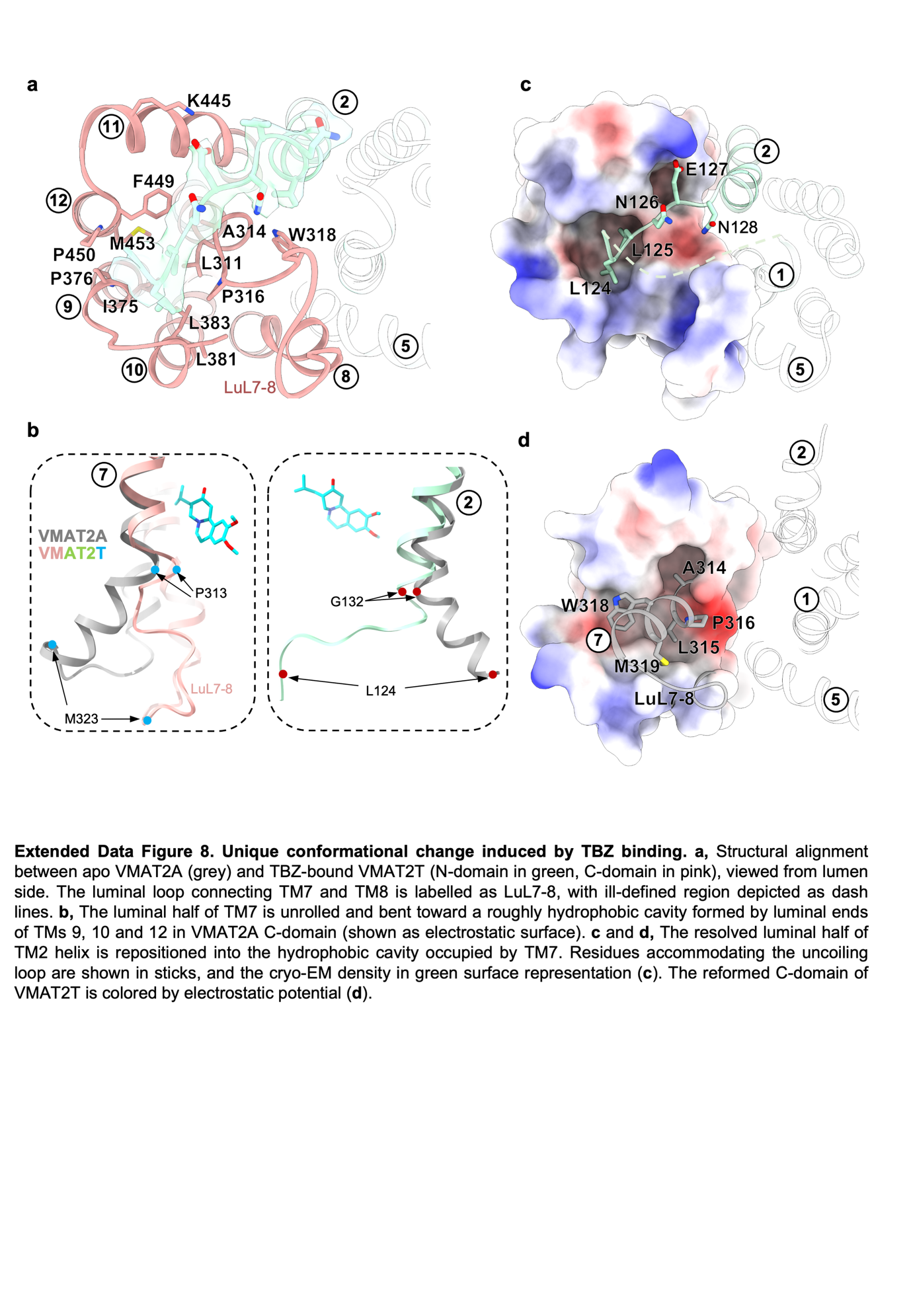


**Fig. S8 Unique conformational change induced by TBZ binding. a,** Structural alignment between apo VMAT2A (grey) and TBZ-bound VMAT2T (N-domain in green, C-domain in pink), viewed from lumen side. The luminal loop connecting TM7 and TM8 is labelled as LuL7-8, with ill-defined region depicted as dash lines. **b,** The luminal half of TM7 is unrolled and bent toward a roughly hydrophobic cavity formed by luminal ends of TMs 9, 10 and 12 in VMAT2A C-domain (shown as electrostatic surface). **c** and **d,** The unraveled luminal half of TM2 helix is repositioned into the hydrophobic cavity occupied by TM7. Residues accommodating the uncoiling loop are shown in sticks, and the cryo-EM density in green surface representation (**c**). The reformed C-domain of VMAT2T is colored by electrostatic potential (**d**).
