# Supplementary material for: Transport and inhibition mechanism for VMAT2-mediated synaptic vesicle loading of monoamines
Source: Cell Res. 2024 Jan 2;34(1):47–57. doi: 10.1038/s41422-023-00906-z (PMC10770148; doi:10.1038/s41422-023-00906-z)
Supplement: Supplementary file 9 — Supplementary information, Fig S9 [file 41422_2023_906_MOESM9_ESM.docx]

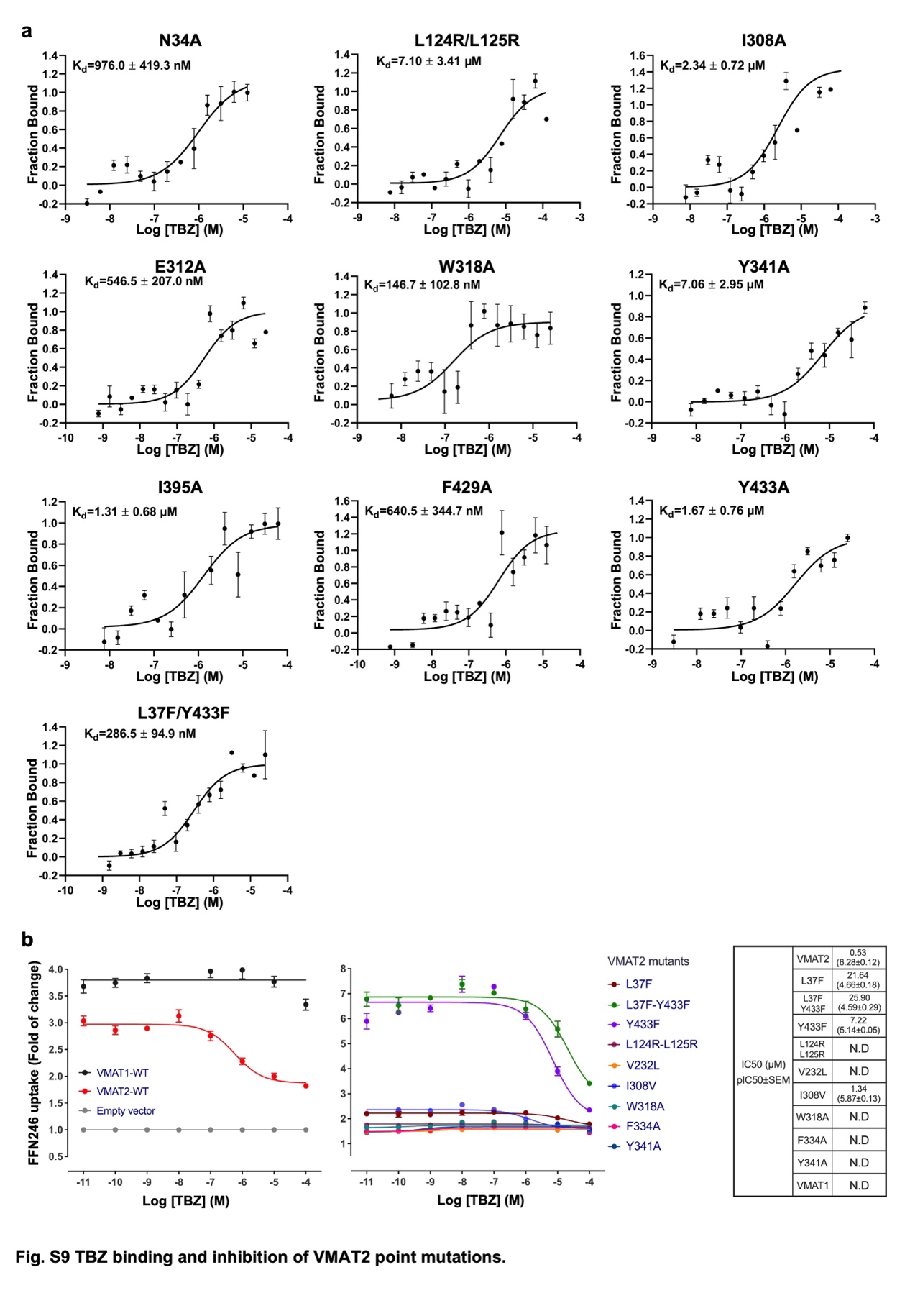


**Fig. S9. TBZ binding and FFN246 uptake of VMAT2 mutations. a,** Affinity of each VMAT2 variant is measured by microscale thermophoresis assay, and displayed with calculated *K*d value accordingly (mean ± SEM, *n*=3-4 independent experiments). **b,** FFN246 uptake activity of VMAT2 mutants affected by TBZ. Concentration-response curves for TBZ inhibition are plotted in different VMAT2 variants and WT VMAT1. IC_50_ for each measurement is summarized in right table. In all panels, error bars represent SEM. N.D: the inhibitory effect of TBZ on VMAT2 mutant is not detectable.
