# Supplementary material for: Transport and inhibition mechanism for VMAT2-mediated synaptic vesicle loading of monoamines
Source: Cell Res. 2024 Jan 2;34(1):47–57. doi: 10.1038/s41422-023-00906-z (PMC10770148; doi:10.1038/s41422-023-00906-z)
Supplement: Supplementary file 10 — Supplementary information, Fig S10 [file 41422_2023_906_MOESM10_ESM.docx]

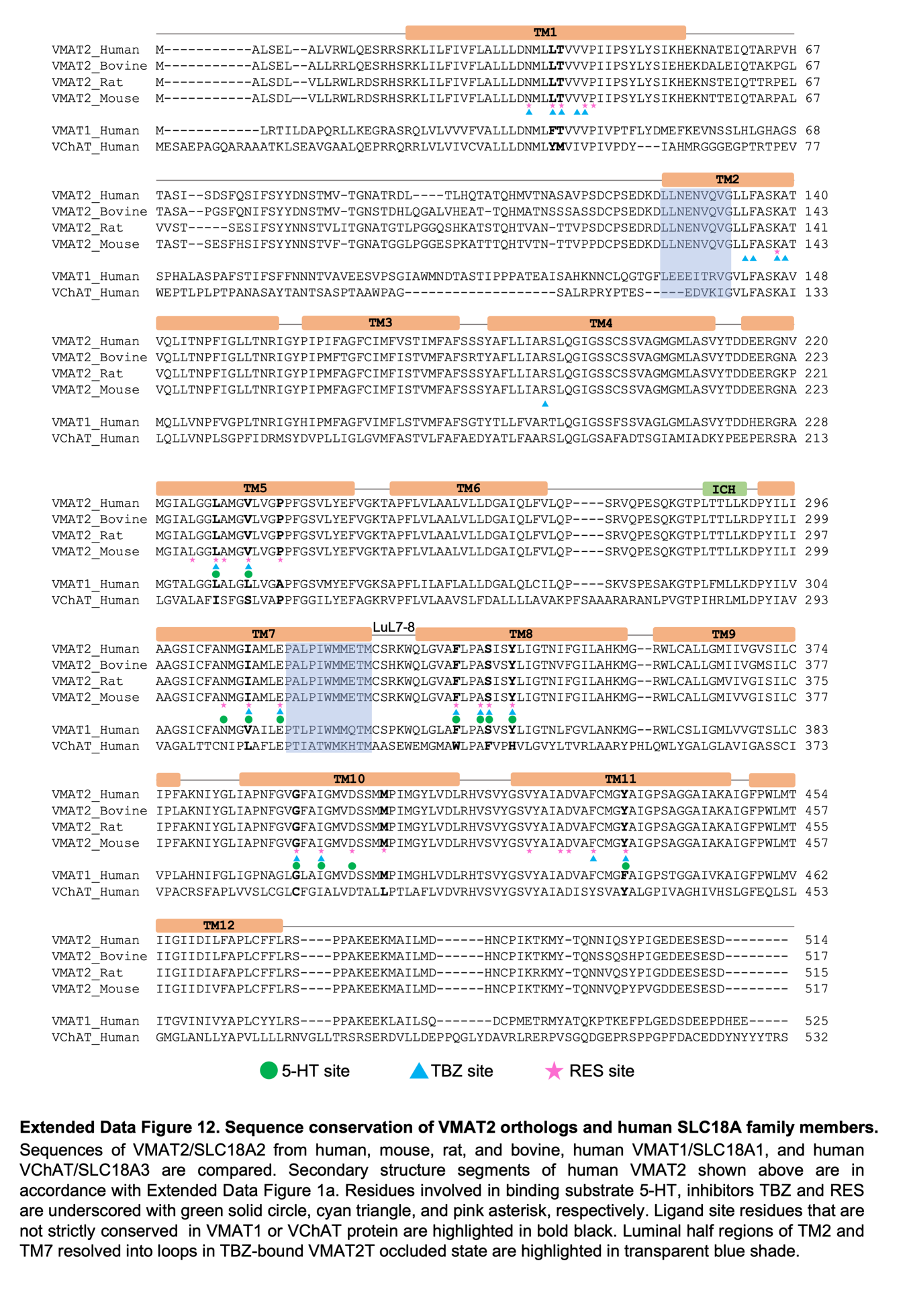


**Fig. S10. Sequence conservation of VMAT2 orthologs and human SLC18A family members.** Sequences of VMAT2/SLC18A2 from human, mouse, rat, and bovine, human VMAT1/SLC18A1, and human VChAT/SLC18A3 are compared. Secondary structure segments of human VMAT2 shown above are in accordance with Extended Data Figure 1a. Residues involved in binding substrate 5-HT, inhibitors TBZ and RES are underscored with green solid circle, cyan triangle, and pink asterisk, respectively. Ligand site residues that are not strictly conserved in VMAT1 or VChAT protein are highlighted in bold black. Helical regions of TM2 and TM7 at luminal side resolved into loops seen in TBZ-bound VMAT2T occluded state are highlighted in transparent blue shade.
