# Supplementary material for: Transport and inhibition mechanism for VMAT2-mediated synaptic vesicle loading of monoamines
Source: Cell Res. 2024 Jan 2;34(1):47–57. doi: 10.1038/s41422-023-00906-z (PMC10770148; doi:10.1038/s41422-023-00906-z)
Supplement: Supplementary file 11 — Supplementary information, Fig S11 [file 41422_2023_906_MOESM11_ESM.docx]

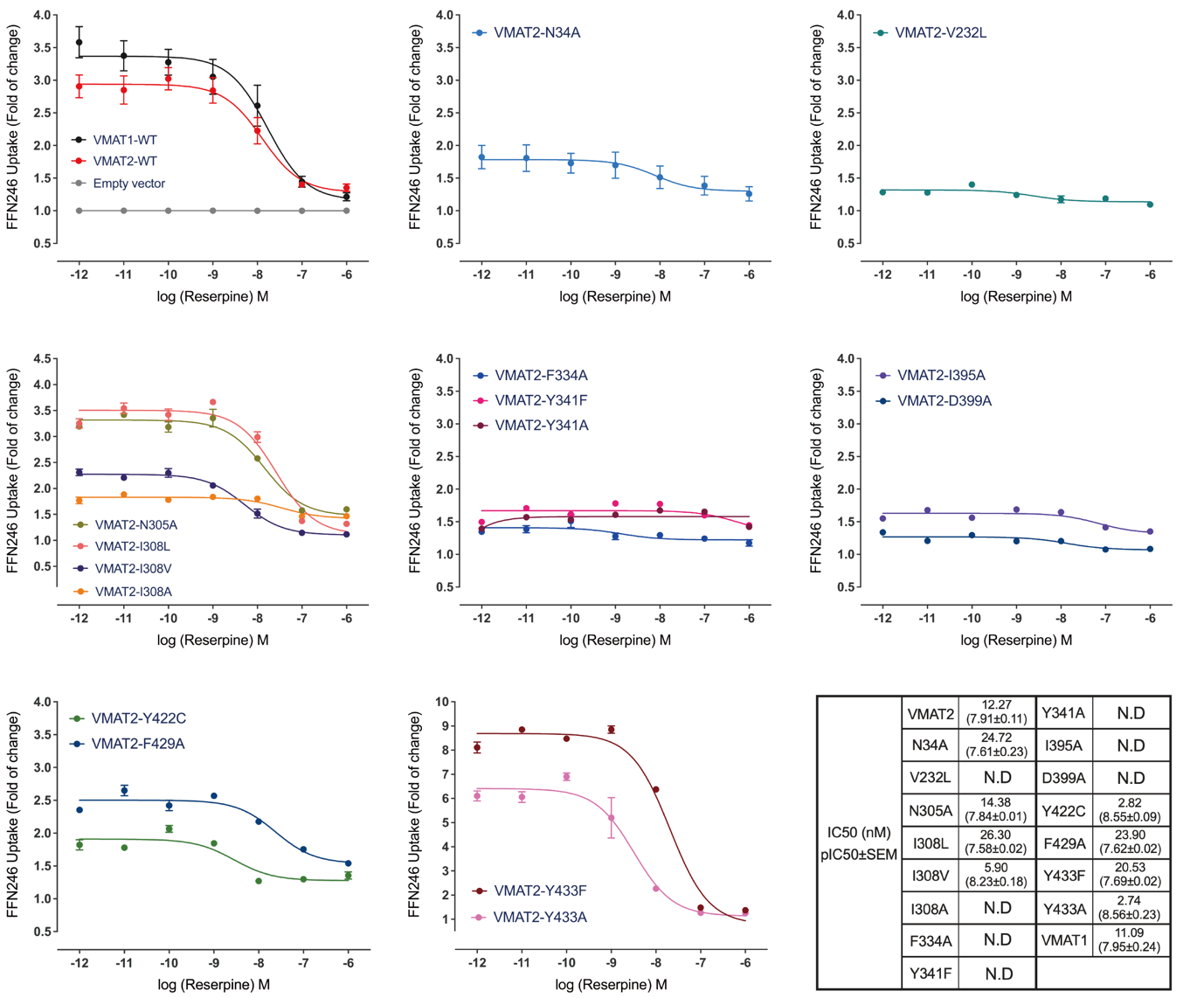


**Fig. S11 FFN246 uptake activity of VMAT2 mutants inhibited by RES.** Concentration-response curves for reserpine inhibition are plotted in different VMAT2 variants and WT VMAT1 protein. IC_50_ for each measurement is summarized in table at the right corner. In all panels, error bars represent SEM. N.D: the inhibitory effect of RES on VMAT2 mutant is not detectable.
