# Supplementary material for: Transport and inhibition mechanism for VMAT2-mediated synaptic vesicle loading of monoamines
Source: Cell Res. 2024 Jan 2;34(1):47–57. doi: 10.1038/s41422-023-00906-z (PMC10770148; doi:10.1038/s41422-023-00906-z)
Supplement: Supplementary file 12 — Supplementary information, Table S1 [file 41422_2023_906_MOESM12_ESM.docx]

|  | **VMAT2A** | **VMAT2S** | **VMAT2T** | **VMAT2_YC_R** | **VMAT2R** | **VMAT2_YC_S** |
| --- | --- | --- | --- | --- | --- | --- |
| **Data collection and processing** |  |  |  |  |  |  |
| Magnification | 130,000 | 130,000 | 130,000 | 105,000 | 130,000 | 130,000 |
| Voltage (kV) | 300 | 300 | 300 | 300 | 300 | 300 |
| Electron exposure (e^-^/Å^2^) | 50 | 50 | 50 | 60 | 50 | 50 |
| Defocus range (μm) | -1.2 to -2.0 | -1.2 to -2.0 | -1.2 to -2.0 | -1.2 to -2.0 | -1.2 to -2.0 | -1.2 to -2.0 |
| Pixel size (Å) | 0.932 | 0.932 | 0.932 | 0.832 | 0.932 | 0.932 |
| Symmetry | C1 | C1 | C1 | C1 | C1 | C1 |
| Initial particle (no.) | 21,827,845 | 10,943,366 | 21,933,726 | 9,162,145 | 8,579, 107 | 5,939,244 |
| Final particle (no.) | 220,731 | 124,189 | 335,823 | 132,817 | 166,931 | 175,647 |
| Map resolution (Å) | 3.60 | 3.57 | 3.37 | 3.74 | 4.75 | 4.10 |
| **Refinement** |  |  |  |  |  |  |
| Initial model used (PDB code) | AlphaFold2 model | 8WLJ | 8WLJ | 8WLJ |  |  |
| Map sharpening *B* factor (Å^2^) | 202.5 | 199.2 | 200.3 | 217.6 | 385.8 | 274.8 |
| Model composition |  |  |  |  |  |  |
| non-hydrogen atoms | 4,035 | 2,804 | 2,873 | 1,815 |  |  |
| Protein residues | 530 | 382 | 383 | 389 |  |  |
| Ligands |  | 1 | 1 | 1 |  |  |
| *B* factor (Å^2^) |  |  |  |  |  |  |
| Protein | 117.55 | 109.41 | 95.79 | 101.62 |  |  |
| Ligand |  | 100.51 | 91.78 | 82.44 |  |  |
| R.m.s. deviations |  |  |  |  |  |  |
| Bond lengths (Å) | 0.004 | 0.004 | 0.004 | 0.005 |  |  |
| Bond angles (°) | 0.762 | 0.789 | 0.856 | 0.940 |  |  |
| Validation |  |  |  |  |  |  |
| MolProbity score | 1.50 | 1.74 | 1.62 | 1.63 |  |  |
| Clash score | 7.96 | 9.84 | 9.42 | 8.90 |  |  |
| Poor rotamers (%) | 0.00 | 0.00 | 0.00 | 0.00 |  |  |
| Ramachandran plot |  |  |  |  |  |  |
| Favored (%) | 97.71 | 96.56 | 97.36 | 97.14 |  |  |
| Allowed (%) | 2.29 | 3.44 | 2.64 | 2.86 |  |  |
| Outliers (%) | 0.00 | 0.00 | 0.00 | 0.00 |  |  |
| Deposited model | 8WLJ | 8WLM | 8WLK | 8WLL |  |  |
| Deposited map | EMD-37621 | EMD-37624 | EMD-37622 | EMD-37623 | EMD-37623 | EMD-37623 |

**Supplementary information, Table S1. Cryo-EM data collection and refinement statistics.**
