# Supplementary material for: Transport and inhibition mechanism for VMAT2-mediated synaptic vesicle loading of monoamines
Source: Cell Res. 2024 Jan 2;34(1):47–57. doi: 10.1038/s41422-023-00906-z (PMC10770148; doi:10.1038/s41422-023-00906-z)
Supplement: Supplementary file 13 — Supplementary information, Table S2 [file 41422_2023_906_MOESM13_ESM.docx]

| Systems | Protein conformation | Substrate | No. of  POPC | No. of  Water | No. of  Na+ | No. of  Cl- | Total  atoms | Replicas | Simulation  Time (ns) |
| --- | --- | --- | --- | --- | --- | --- | --- | --- | --- |
| VMAT2-5HT | Cytosol-facing | 5HT | 120 | 10527 | 26 | 33 | 53686 | 3 | 500 |
| VMAT2-TBZ | Occluded | TBZ | 120 | 10545 | 27 | 31 | 53750 | 3 | 500 |
| VMAT2-RES | lumen-facing | RES | 118 | 10428 | 27 | 32 | 53296 | 3 | 500 |
| VMAT2-5HT  (D399-Protonated) | Cytosol-facing | 5HT | 120 | 10223 | 26 | 34 | 52777 | 3 | 500 |
| VMAT2-5HT  (E312-D399-Protonated) | Cytosol-facing | 5HT | 120 | 10212 | 26 | 35 | 52638 | 3 | 500 |

**Supplementary information, Table S2. Parameters for MD simulation system setup.**
